# Big Data Analytics, Infectious Diseases and Associated Ethical Impacts

**DOI:** 10.1007/s13347-017-0278-y

**Published:** 2017-08-24

**Authors:** Chiara Garattini, Jade Raffle, Dewi N Aisyah, Felicity Sartain, Zisis Kozlakidis

**Affiliations:** 1 Anthropology and UX Research, Health and Life Sciences, Intel, London, UK; 2 Division of Infection and Immunity, University College London, Cruciform Building, Gower Street, London, WC1E 6BT UK; 3 Department of Infectious Disease Informatics, University College London, Farr Institute of Health Informatics Research, 222 Euston Road, London, NW1 2DA UK; 4 Profiscio Ltd., 10 Great Russell Street, London, WC1B 3BQ UK

**Keywords:** Infectious diseases, Big data analytics, Ethics, Mobile phones, Wearables

## Abstract

The exponential accumulation, processing and accrual of big data in healthcare are only possible through an equally rapidly evolving field of big data analytics. The latter offers the capacity to rationalize, understand and use big data to serve many different purposes, from improved services modelling to prediction of treatment outcomes, to greater patient and disease stratification. In the area of infectious diseases, the application of big data analytics has introduced a number of changes in the information accumulation models. These are discussed by comparing the traditional and new models of data accumulation. Big data analytics is fast becoming a crucial component for the modelling of transmission—aiding infection control measures and policies—emergency response analyses required during local or international outbreaks. However, the application of big data analytics in infectious diseases is coupled with a number of ethical impacts. Four key areas are discussed in this paper: (i) automation and algorithmic reliance impacting freedom of choice, (ii) big data analytics complexity impacting informed consent, (iii) reliance on profiling impacting individual and group identities and justice/fair access and (iv) increased surveillance and population intervention capabilities impacting behavioural norms and practices. Furthermore, the extension of big data analytics to include information derived from personal devices, such as mobile phones and wearables as part of infectious disease frameworks in the near future and their potential ethical impacts are discussed. Considered together, the need for a constructive and transparent inclusion of ethical questioning in this rapidly evolving field becomes an increasing necessity in order to provide a moral foundation for the societal acceptance and responsible development of the technological advancement.

## Introduction

The terms big data and big data analytics are often used within the context of healthcare as an all-encompassing phrase referring to the use of large datasets. Their increasingly regular use does little to signify the underlying complexity of definitions, and paves the way for potential subsequent ethical and social misunderstandings (Floridi 2012). Big data is defined as collections of data so large and highly complex that its manipulation and management require the application of a series of computing techniques—including but not limited to—machine learning and artificial intelligence (Stuart Ward and Barker 2013). Big data analytics is defined as ‘the process of collecting, organizing and analysing large sets of data (called big data) to discover patterns and other useful information’ (Heymann and Rodier 2004). Within the sphere of routine healthcare and associated research, the term is often synonymous to electronic patient records from central public authorities, large hospitals, clinical trials, ‘-omics’ and sequencing-based outputs and their associated banked samples, imaging, mobile phones and/or wearables. These data can be structured or unstructured, generated from diverse sources and at diverse speed, and in very large volumes. Big data analytics (BDA) are the collectively termed processes that translate the big data into interpretable and potentially actionable sets of information that can confer a competitive advantage (LaValle and Lesser 2011).

BDA have attracted attention as they have been shown to have transformational potential for generating reliable, actionable and novel insights about the world we live in. Positive examples have been reported in the financial services (Mayer-Schoenberger and Cukier 2013) and in healthcare (Murdoch and Detsky 2013; Raghupathi and Raghupathi 2014; Lee et al. 2016). For infectious diseases, BDA is useful in monitoring diseases outbreaks; stratifying patients for treatment, risk exposure and treatment outcome prediction; and for the prediction of behavioural patterns with the aim of informing public health interventions (Darrell et al. 2015; Lee et al. 2016). Despite these positive outcomes, the routine use of BDA is increasingly challenged with examples of wrongful conclusions (Lazer et al. 2014), potential misuse of personal information, well-publicised privacy breeches and ongoing profiling of individuals for commercial purposes (Smith 2012; Richards and King 2014; Martin 2015). Often described as unintended (Wigan and Clarke 2013), these are very real risks (Clarke 2016). However, these risks are not always predictable or preventable, as the context in which big data is used heavily determines which of those problems actually occur and whether they may lead to negative consequences at the individual and/or societal levels (Markus 2015; Zuboff 2015; Duhigg 2012).

In the field of medical ethics, the basic principles of autonomy, beneficence, non-maleficence and justice remain relevant for BDA (Gillon 1994; Page 2012). However, they are tested by the differential interpretation relating to their contextual application in clinical care, public health policy, administrative repurposing, patient-generated data and research (e.g. clinical trials and/or use of metadata) with BDA acting as a magnifying glass for potential differences and difficulties (Bollier 2010). Furthermore, the way in which data have increasingly been collected outside of traditional healthcare settings and shared with third parties for research and commercial gains has also changed (Jetten and Sharon 2016). These factors are expected to intensify further as data collection is expected to scale up and become more distributed through ‘non-traditional’ channels with its processing being quicker and more automated.

Given these potential issues associated with BDA and considering its increased uptake, discussions are ongoing regarding what constitutes its ethical versus unethical use in healthcare. There are a number of studies addressing this aspect (Mittelstadt and Floridi 2016), which is particularly pertinent in the field of infectious diseases, where the impact of BDA is already tangible and is further augmented by the ‘non-traditional’ data collection systems, such as wearables and mobile phones (Vanhems et al. 2013). In this paper, we discuss some of the changes occurring in the field of BDA in infectious diseases by comparing the traditional model of data accumulation to the new one. We then discuss the ethical challenges according to four headings: (i) automation and algorithmic reliance impacting freedom of choice, (ii) big data analytics complexity impacting informed consent, (iii) reliance on profiling impacting individual and group identities and justice/fair access and (iv) increased surveillance and population intervention capabilities impacting behavioural norms and practices.

## Information Accumulation Models in Infectious Diseases and the Impact of BDA

The traditional information accumulation model in infectious diseases remains a variant of a hierarchical hub-and-spoke model, where well-defined smaller reporting centres (such as GP practices or individual virologists) report to a central authority at the local level (such as referral hospitals) or at the national level (such as Public Health England in the UK) for the epidemiological view. The accumulated information is aggregated at population level, processed, corrected/defined and processed again (in a number of iterative cycles). Subsequently, actions relating to these conclusions are disseminated to the whole system in a top-down approach. This is economical in the sense of using defined communication channels according to established professional and well-regulated processes, recommendations, actors and actions. In addition, there are bona fide population data aggregators, such as national health surveys, administrative hospital data and biobanks, at a local national or international level, for example the UK Biobank in the UK and the BBMRI-ERIC in the European Union (Biobanking and Biomolecular Resources research infrastructure–European Research Infrastructure Consortium) (Kozlakidis 2016).

However, the information accumulation and processing steps carry an inevitable time lag that can result in reduced response effectiveness at a public health intervention level. The usually slow methods of assessing medical knowledge through the accumulation of evidence-based cases are challenged by rapidly evolving global threats. This can have catastrophic results when dealing with infectious disease occurrences as was observed in the case of the recent Ebola outbreak in West Africa (McCarthy 2016) or in the cases of the global influenza pandemics in 2008–2009 (Doshi 2009). It should be noted though that the established, global hub-and-spoke system is still considered successful in identifying potential disease outbreaks at an international level (Heyman and Rodier 2001; Heyman and Rodier 2004) and some national levels (Randrianasolo 2010). The need for speed in data sharing/accumulation, processing and advice provision in the case of unexpected/novel outbreaks was identified early on and became one of the main drivers for introducing BDA capabilities. The initial piloted learnings were then distilled and used as improvements on the hub-and-spoke systems (Jacobsen et al. 2016; Simonsen et al. 2016).

The above information accumulation models are most effective in the cases of sedentary, well-defined, well-predictable populations and outbreaks. They do not necessarily reflect the current global contextual complexity or the patchy and/or often unstructured nature of real-time data. As such, they come under increasing stress and criticism for lack of adaptability. Mass migrations—often resulting from emergencies such as floods and wars—can produce conditions that exacerbate this phenomenon, spreading infectious diseases, often in unexpected ways. Global trade, mass gatherings and travel can introduce pathogens into new populations creating the potential to develop into epidemics, e.g. West Nile virus introduction and spread in New York (Fonkwo 2008), post-festival measles outbreak in Germany (Pfaff et al. 2010) and H1N1 spread through airline transportation (Khan et al. 2009).

### New Sources of Information Accumulation

Mobile phones can be used to address global and healthcare data inequities and hold particular promise in low- and middle-income countries (LMIC) where conventional sources of social and health-related data are often patchy, out of date or simply non-existent (Kaplan 2006; Deville et al. 2014; Center for Global Development 2014). The extraordinary reach and potential effectiveness of engaging mobile technologies was demonstrated by (a) an emergency reporting system for infectious diseases surveillance in Sichuan province, China, following the catastrophic earthquake of 2008 (Yang et al. 2009); (b) a monitoring system for the long-distance observation of tuberculosis patients in Kenya (Hoffman et al. 2010) and (c) a guidance tool for potential Ebola patients providing directions to the closest available health centres (Trad et al. 2015). In the UK, a mobile phone-enabled, video-observed therapy clinical trial for adherence to treatment for tuberculosis patients is already underway (Story et al. 2016) and under consideration as part of an increased patient offering by the UK public health system. In addition, a number of mobile-enabled infectious diseases diagnostic tools are currently under development. The use of mobile phone technologies as part of mainstream healthcare is considered largely inevitable (Dell et al. 2011).

There are many studies describing the development of different sensor technologies and wireless medical instruments (collectively termed as wearables) with the ability to remotely monitor patients at a distance, at home or elsewhere, by recording personal parameters such as blood pressure, heart rate, sugar levels, vital signs, oxygen levels, body temperature, and mental well-being metrics (Chana 2012). The main driver behind these developments remains the need for the early warnings for the onset of adverse health conditions and service improvements coupled with costs reductions in the health sector (Page 2015a). Wearable devices can transmit vast amounts of data sometimes in constant live streaming modes which raises important ethical issues regarding privacy and security. Individuals, organisations and businesses can be severely affected if third parties access private information in an unlawful manner. Although wearable devices are designed to promote independence, they require different levels of privacy intrusion to collect data, especially when recording passively, therefore making it important to consider ethical implications from different stakeholders’ perspectives (Yeslam 2015). Additionally, choice/autonomy and security/privacy risks can be bidirectional, i.e. from pre-selected incoming data (i) equipment might be adjusted to allow for greater provider preference as opposed to patient preference, in the case of medication pre-order for example and (ii) risks can arise from the outgoing information, as well as by incoming information into the device, as malware can jeopardize patients’ health by causing some (intentional or unintentional) malfunction of the device (Segura et al. 2017).

### BDA Impact on Infectious Diseases

The most commonly mentioned impact of BDA in the field of infectious diseases relates to the access and use of information contained in individual medical records and/or collected using healthcare systems’ channels. In this context, insights generated by routine access and use of these data, including when explicit consent each time is not a viable option (such as in the case of large-scale, multiple-site epidemiological studies), can potentially benefit patients, institutions, public health as well as commercial entities. The main difference with the traditional regime relates to the scale and reach of BDA capabilities, accessing and processing much larger numbers of medical records and more information per record. However, the levels of access and use of the information do vary between locations and for different purposes. In the field of infectious diseases, decision is guided by two separate specialties’ viewpoints: epidemiology, looking at the overall population-level health, and infectious diseases specialties (such as microbiology, virology) addressing the individual-level health. This is reflected in the ethical arguments framed as autonomy versus public good, where BDA has acted as a catalyst in the arguments on either side (Gilbert 2012; Kozlakidis et al. 2012; Blais and White 2015).

From an individual perspective, the availability of vast amounts of information regarding people, for example via geo-tagged social networks, makes even data anonymization ineffective in fully protecting the identity of the data source, making it only more difficult, yet still feasible via triangulation, to (re)identify it (Cecaj et al. 2016). As such, the ethical imperative of transparency with regard to the dangers of downstream data linkage and inadvertent individual identification should be upheld. To this end, the Groupe Speciale Mobile Association (GSMA) has developed guidelines for the appropriate use of Call Detail Record (CDR) and Standard Messaging Services (SMS) data in emergency situations (GSMA 2013) and updated them specifically for infectious diseases based on the experiences from the Ebola outbreak in West Africa (GSMA 2014) with some limited but not extensive consideration of potential ethical implications.

From a population-level perspective, if systems are designed to rely entirely on anonymous contributions in order to protect their original data contributors, they might not work well either, as the element of information accountability and, hence, transparency is affected. Especially in the case of humanitarian emergencies, and certainly communicable disease outbreaks, anonymous information can be accompanied by a climate of fear and mistrust. Information should ideally be provided with the source location and time for full accountability and to aid appropriate infection control (Bayham et al. 2015; Funk et al. 2009). The possibility of malevolence under such a scenario, for example by spreading rumours (intentional or unintentional), has to be taken seriously. Anonymous mobile-enabled reporting systems within these contexts could result both in poor quality data, and in the provision of a platform for increased community disharmony (Cinnamon et al. 2016).

## The BDA Impact on Infectious Diseases Ethics

The allure of these technological applications and their impact is coupled with as yet unanswered ethical challenges. BDA-enabled infectious diseases tools could exacerbate the tensions between individual and public good. A number of these ethical questions are not novel; however, the scale at which they can occur and their potential impact has now been multiplied, as a small number of decisions/actions can now potentially affect multiple populations at different geographical locations at the same time. At what point are individuals prepared to compromise on some rights, such as that of privacy and autonomy, if it benefits them? Under what circumstances in the field of infectious diseases can individuals give up some individual benefits in order to greatly enhance public good? And, assuming that there is a benefit to the individual directly (e.g. appropriate medical treatment through better tailored response), to what extent should society be able to operate without disclosure and transparency regarding decisions taken automatically through data access and use?

### Freedom of Choice

One of the key challenges in the field of BDA in infectious diseases is connected to the difficulty for individuals to be fully aware of what happens to their data after collection, as this might be generated in one locality, aggregated in a second, processed in a third, and so on. In other words, the initial data often moves through an information value chain: from data collectors, to data aggregators, to analysers/advisors, to policy makers, to implementers. Data aggregators and analysers combine the data from multiple sources and create a new picture of individuals based on the data received (Bollier 2010). Ethical issues can arise for each segment of this collection and distribution chain, even without utilizing any BDA on the data at all, with the final actor/implementer using the data for purposes that can be very different from the initial intention of the individual that provided the data.

As more data is used at increasing scale, there are growing concerns relating to aspects of automation, which is often associated to erosion of autonomy and a reduction in personal choices (Hildebrandt and Rouvroy 2011). Machine learning tries to identify relationships within big data sets and subsequently, the context in which these identified relationships are meaningful. The difficulty in accurately predicting any human behaviours based on such data correlations, lies in the tendency to ignore (or normalize) the human design and inherent error and biases in data, measures and analyses (Zuboff 2015). Furthermore, the tendencies of automation to flatten outliers and confirm patterns, to equate correlation with causation and to make it difficult to judge assumptions (i.e. lack of transparency) are also problematic (Hildebrandt 2011). The ethical question then remains: under what circumstances, if any, are such statistical approximations and reduced personal choices acceptable? The initial use of BDA as decision support can transit into an automated decision-making process, especially if the decision support uses have proven medically effective. Perhaps this is an opportunity to investigate technical mechanisms that can introduce an element of human oversight to inform and safeguard on the ethical aspects of automation, decision support and decision-making. In particular, the freedom of choice to opt-out or disagree with both decision support and decision-making outcomes should be coupled with the ability to request a second opinion that is entirely independent of the automated mechanism (e.g. an independent specialist consultation) and without any potential implications on future healthcare provision. In this manner, individuals are guaranteed a choice within existing norms and without future penalties.

### Informed Consent

The reliance on algorithms for analyses in infectious diseases can introduce a gradual reduction of general understanding of the decision-making process, as the latter becomes an indecipherable black box (Pasquale 2015). Recognizing BDA as a complex process with several contributing actors including private firms is therefore important and requires transparency at each step (Asadi Someh et al. 2016; Martin 2015). Is it then ethical, or even at all possible, to achieve a truly informed consent in everyday routine clinical practice and public health, on systemic data usage in the era of BDA? Or should the model of informed consent for BDA-enabled data usage be applied fully, only to research data and clinical trials?

In an effort to navigate through some of these complexities and in the wake of the influenza pandemic, the WHO has advised that there must be a clear distinction between the boundaries of public-health oriented research and clinical practice. The informed consent of clinical practice carries the well-defined risk of preceding evidence-based practice, while the research-based activities carry a higher risk which should be made clear during the consenting process. The implementation of BDA with the potential for reduced understanding of the process also carries the risk of obfuscating the understanding of risk during consenting. Distinguishing between clinical research and practice is important, even if it is often difficult, because of the varied ways in which they are perceived and regulated in different countries (WHO 2007). The practicality of this recommendation was severely tested in the recent Ebola outbreak, where BDA-enabled research and clinical decision-making had to be concurrent due to the nature and timings of the outbreak (WHOERT 2014).

In the case of viral diagnostics for example, the amount and granularity of information provides not only the knowledge regarding potential drug resistance parameters by the infecting organism but also the reconstruction of infectious disease outbreaks, transforming the question of ‘who infected whom’ into ‘they infected them’, i.e. from the more general to the definitive form (Escobar-Gutierrez et al. 2012; Pak and Kasarskis 2015; Bosch et al. 2016). In this context, the informed consent should be able to articulate the potential for group-level identification of infectious diseases through BDA. Most informed consent forms used currently in clinical practice identify personal risks and ignore group-level risks, perhaps as the latter are more challenging to identify and quantify.

### Profiling and Justice

In order to create actionable and interpretable recommendations, aggregated population data are used to stratify the population into smaller groups to support realistic public health interventions. This method is called profiling and can occur by classifying individuals into groups, based on any given characteristic, including race, ethnic group, gender, and socio-economic status (LaBeaud et al. 2008; Newell and Marabelli 2015). However, the patient profiling in the field of infectious diseases is not always as straightforward. An important ethical aspect is the transition of an infected patient, from a vector of a disease to a potential victim, to a potential transmission node. Although technically, the link between these states can now be proven unequivocally and at increasing scale through modern diagnostics, there is a paucity on social science and ethics research exploring how these patients understand and react to these transitions—and in relation to therapeutic interventions (established or experimental). How can this status transition be ethically accounted for in the BDA era? A consent form for infectious disease research should reflect this transition and explain the context-dependent nature of the terms that are used in research. BDA should be able to accommodate the complexity arising from the possibility for patients to appear with a perceived duality of status (infecting and infected) at the same time, both at the individual and the population level.

Furthermore, the algorithms that enable profiling can ignore outliers (e.g. by reverting to mean) and provide the basis for (intentional or unintentional) discrimination among individuals or groups by downstream policy makers and implementers. As profiling (or at least some approximation) is inevitable—it is after all a very practical approach in order to rationalize the vast amounts of information into actionable information—how can individuals and group identities and differentiators be protected? Can there be safeguards in place to protect the predictable becoming exploitable? In order to achieve fairness and transparency, individuals should be made aware that decisions were taken by including profiling algorithms. There should be provision for meaningful information on the BDA approaches used and the assurance that the individual view point can be expressed, that a challenge of the decision-making through profiling will not impact future healthcare provision and that an alternative human intervention can be provided.

### Surveillance and Behaviour

If effective infectious disease control policies are to be implemented in a given population, the surveillance of outbreaks needs to be coupled through BDA with modelling that predicts an individual’s or a group’s behaviour. Healthcare organizations can have the technological ability for the first time to continuously observe and monitor behaviours through mobile phone apps or wearable devices, offering personalized services and advice, which may imply that these individuals are no longer exposed to all options and choices that would normally be available. Is the pre-defined reduction of free choice acceptable when coupled by more effective (in terms of treatment outcome) and efficient healthcare options? This can only be decided on a case by case basis, where the reduction of free choice is coupled by specific measure to safeguard fundamental rights and the interests of the individual.

There is a substantial body of work relating to the factors that can influence population behaviour in the field of infectious diseases, using specific language to influence emotions leveraging anticipated behaviours, such as higher uptake of vaccination or reduction of contact in public spaces (Bayham et al. 2015; Chapman and Coups 2006)—and how altered social behaviour can then make a real difference in the transmission of an infectious disease within a given population (Kucharski et al. 2014; Funk et al. 2009). The impact of BDA is only expected to provide more and better calibrated opportunities to influence behaviour. Is it then ethical to control or influence an individual’s or group’s behaviour using information derived from their healthcare-related big data? The response to this question would inevitably be context specific and would relate to the relative balance between substantial public interest and potential harm. The proportionate nature of the pursued aim should be made transparent—even if that includes a high level of uncertainty, such as in the case of the recent Zika virus outbreaks in South America.

Additionally, the need for quick actionable advice close to real-time, (e.g. during international sports events; see Schenkel et al. 2006; Franke et al. 2006), regular cultural events, (e.g. during the Hajj; see Memish 2009) or routine operations (e.g. hospital emergency departments admissions; see Muscatello et al. 2005; Heffernan et al. 2004) complicate things further. Complexities that involve real-time predictive modelling are being addressed for unexpected and difficult to predict patterns, (e.g. climatic conditions; see Sultan 2005; Keller et al. 2009) or one-off and high-impact events (e.g. potential bioterrorist acts; see Kortepeter et al. 2000; Buehler et al. 2003). Recently, the effects of novel computing techniques and implementations have started to emerge. Algorithms can provide automated decision support as part of clinical workflow at the time and location of the decision-making, without requiring clinician initiative, and hence leading to cost and service improvements (Darrell et al. 2015). For example, as part of implementing hospital-wide analytical decision-making tools, patients presenting in emergency rooms are entered into an electronic health records system, where an algorithm can evaluate their suitability for seasonal flu vaccinations. This, in turn, prompts clinical staff to offer it, leading to a greater uptake of the vaccine and downstream savings (Venkat et al. 2010). The possibility of implementing real-time, responsive and adaptive calculations has great potential and is therefore very tempting. However, because of its direct impact on the provision of care and its resourcing in sometimes unpredictable ways, as well as its automated nature, it presents ethicists and regulatory bodies with challenges. These centre on the potential pressures of reduced autonomy and choice, the difficulties of obtaining consent or potential for automated ‘lack of consent’ policies and even, as a worst-case scenario, the potential for unintentional harm.

## Considerations and Recommendations

BDA has the potential to act as a catalyst and transformative agent in healthcare introducing a new era of data utilization, particularly in the field of infectious diseases. However, the ethical implications are not fully explored and if not addressed might become limiting factors preventing BDA from reaching its full potential. There are three further aspects that will be considered in this section, relating to the future developments of the BDA applications in the field of infectious diseases.

### Transdisciplinary View of BDA Ethics Impact

A large number of studies on BDA in healthcare reflect the uni-professional legacy of medical training and funding, yet there is a call for more collaboration between medical, life and computational sciences—often termed as ‘convergent’ or ‘inter-professional’ approach (Sharp et al. 2016; Nelson and Staggers, 2017). Such ‘convergence’ has obvious potential for new discoveries. It also requires the integration of historically distinct disciplines, technologies and ethical viewpoints into a new unified whole. The further development of BDA ethics in infectious disease will need to include an understanding of different users’ needs and capabilities, of the technologies and of the problems investigated. BDA can accommodate and perhaps be even more effective if studies conducted were based on transdisciplinary and participatory consultation designs, especially because there are so many questions yet to be defined and researched.

For example, one could conduct studies where patients would have the right to access their data, see their diagnosis/prognosis and have the ability to add their response to it (therefore both meant to collect data and intervene at the same time). These patient responses can then be used as validation inputs for BDA-based patient profiling, with the patient once again having the opportunity to view this information, applying a consistent, transparent approach throughout the patient contact points. A number of such initiatives have already taken place at different areas of the developed world. However, the usefulness of this approach is still hard to quantify as the outcome metrics used were implemented within short time frames. Additionally, there is a lack of rigorous empirical testing that can separate the effect of record access from other existing disease management programs (de Lusignan et al. 2014; Jilka et al. 2015).

However, it should be noted that the traditional metrics used for the impact of infectious diseases outbreaks still measure and report to a large extent the immediate medical impact of outbreaks—not the wider socio-economic implications. This is done for a number of reasons, such as the available infrastructure, the availability of skilled labour and the existing reporting structures, which are not able to measure the wider impact of infectious diseases outbreaks. (Heyman et al. 2015; Kruk et al. 2015) As such, any improvements to traditional approaches—through BDA or other means—do carry a potential multiplier effect in terms of their impact, where a moderate improvement in clinical outcome is still considerable within a wider socioeconomic context. The information provided through this transdisciplinary view can include, inform and improve the ethical arguments made in relation to the public good.

### Ethical Considerations Are a Competitive Advantage

In the case of established infectious disease operations, the speed of technological development and the incorporation of new technologies, such as mobile phones and wearables, have outpaced the adaptability of current frameworks. For example, there is still a very slow uptake within healthcare of mobile phone-enabled appointment systems, even though the technology has existed for over a decade. Especially in the heavily regulated sector of healthcare provision, the speed of development and lack of flexibility demonstrate the need for further work in the field. One example of this type of discourse is the creation and world-wide distribution of thousands of healthcare-related applications. The majority of those are created by individuals or private providers, not institutions, and very few are regulated or if they are, they might conform to local regulations that may conflict within different regions globally. The availability and uptake of applications where individuals may voluntarily input part of their medical record has not been highlighted widely as a potential ethical minefield, even though it presents challenges not only in terms of provision of erroneous information, but also in terms of downstream personal information usage. Mobile phone diagnostics developed by healthcare providers and validated through the implementation of international standards are also present into this highly competitive melee. The FDA does not regulate applications that—in case of malfunction—do not pose a health threat to others. As such, thousands of health-related, data-rich applications remain without any regulation (Husain and Spence 2015). In light of this regulatory gap, addressing the rounded consideration of ethics can serve an acute competitive advantage for the application developer (Gibbs et al. 2016) and as a pre-condition for wider adoption into the healthcare provision system.

### Open Data Policy and Education

The open data movement involves governments and large public and private organizations making many of their datasets publicly available, preferably in structured machine readable formats. The underlying drive for this concept is that individual citizens, private sector and non-governmental organizations will, on a self-service basis, access and exploit these resources. However, the description of each individual is contextually dependent and informationally inexhaustible: i.e. there is no end to the potential use of one’s data once created; data can be generalized, aggregated, defined, refined, repurposed, profiled and so on. The understanding of digital risks and ethical implications is lacking and should become a substantial part of open data or of data collection practices and of digital literacy training courses. As the information for an individual person is aggregated multiple times with information for many other millions of individuals and parameters, and information is extracted at multiple levels, understanding the pathway and processing of data becomes increasingly difficult for individuals to attain. In much the same manner, the attribution of accountability in the case of unintended harm through the same means becomes difficult to ascertain. As such, the recommended approach would be that of (ideally) introducing appropriate permissions for information generators and/or custodians at the point of data release and pursue transparency thereof, so that the information flow can be mapped, followed and its use audited if necessary or possible. Where knowledge dissemination is the only realistic potential benefit, e.g. following infectious disease surveillance audits, researchers’ ethical obligations need to include the understanding and description of potential collective risks in addition to the often mentioned individual identification risks.

In summary, the BDA implementation in the field of infectious diseases seems as an inevitable technological development. However, the long-term, widespread acceptance of this addition to the clinical decision-making process and adoption by patients, clinicians and the society needs to take into account the ethical aspects that are augmented or created through BDA. The need for transdisciplinary approaches to address urgent and/or complex situations should be inclusive of a transdisciplinary view of ethical needs as well. The inclusion of patients into the participatory study design and medical record access has allayed fears so far on the potential unethical use of their data through BDA. However, there are as yet no consensus metrics against which such opinions can be recorded, measured and addressed consistently. The identification of potential ethical risks at an individual and group level through BDA in infectious diseases should be a common feature, including the recognition of the contextual nature of potential ethical risks. The above are mainly viewed through the lens of the public healthcare provision; however, there are equally applicable in the case of private healthcare providers that use BDA. The latter have made great strides to develop data safety features as commercially exploitable competitive advantage and is perhaps equally relevant that the inclusion of ethical considerations can become an additional unique selling point.

## Conclusions

The infectious diseases field’s obvious mission is to provide the best possible care for infected individuals in the most efficient way, reduce the risk of outbreaks occurring and/or control existing ones. The less obvious mission is to develop preparedness for future outbreaks. The latter can be achieved through large -scale modelling and BDA utilization (Rocha and Masuda 2016). BDA in infectious diseases has not been designed as an inclusive tool, rather as a faster enabler of existing tools. The increasing availability of information through many non-traditional channels requires increased inclusivity and transparency along the needs for privacy and security. A significant number of studies have been undertaken in order to solve issues of privacy and security from a technological point of view (Safavi 2014; Camaraa 2015). That said more studies need to take place and define the ethical implication of BDA in infectious diseases in terms of (i) loss of individual autonomy and erosion of freedom of choice in response to population-level benefits and the (ii) inclusion of ethical design in the creation of BDA-enabled application aiding decision-making in healthcare provision. The success of these actions would be the wide understanding for the contextual nature of the BDA-aided interpretation and the relative risk-/benefit-based decision during infectious disease outbreaks, not just by clinicians but by the wider society affected by those outbreaks.

It is apparent that despite the positive impacts and advantages of BDA in the field of infectious diseases, the overall consequences for individuals, groups, healthcare providers and society as a whole remain poorly understood and are an under-researched topic. Importantly, an improved awareness and understanding of the ethical issues and consequences is required to avoid the emergence of a potential negative feedback loop, where misunderstandings and lack of transparency can lead to social rejection, distorted policy and to a lack of acceptance of new technologies—limiting their potential impact for individual and societal well-being.

The surveillance potential, accuracy and immediacy of mobile-enabled technologies need to be better understood, in terms of healthcare and social impacts, including the ways in which this technology might be used for ‘social sorting’, such as the categorization of people according to risk, which can have serious real-world consequences (Lyon 2003). Irrespective of emergencies during infectious disease outbreaks, where perhaps the established state or societal norms may be temporarily suspended, the everyday potential benefits of mobile-enabled data collections need to be better balanced against their potential threats to health, freedom, non-discrimination and privacy.

One should emphasize at this point that what is now considered ‘big data’ might be only small data in decades to come. As such, the ethical pressures are expected to intensify further. The current work has highlighted the impact of BDA on the ethics of infectious diseases, as these might present themselves through established and novel perspectives. As the application of BDA in this field is expanding, further work will be necessary to define and address the ethical questions that will arise, as well as to implement consistent transparency to cultivate public trust in these evolving hyper-complex situations.
